# Unraveling the Variability of Essential Oil Composition in Different Accessions of *Bunium persicum* Collected from Different Temperate Micro-Climates

**DOI:** 10.3390/molecules28052404

**Published:** 2023-03-06

**Authors:** Mudasir Hafiz Khan, Niyaz Ahmad Dar, Bashir Ahmad Alie, Sher Ahmad Dar, Ajaz Ahmad Lone, Ghulam Hassan Mir, Uzma Fayaz, Sajad Ali, Anshika Tyagi, Mohamed A. El-Sheikh, Saleh Alansi

**Affiliations:** 1 Advanced Research Station for Saffron and Seed Spices, SKUAST-Kashmir, Srinagar 192121, India; 2 Dryland Agricultural Research Station, SKUAST-Kashmir, Rangreth, Srinagar 191111, India; 3 Department of Biotechnology, Yeungnam University, Gyeongsan 38541, Republic of Korea; 4 Botany and Microbiology Department, College of Science, King Saud University, Riyadh 11451, Saudi Arabia

**Keywords:** essential oils, GC-MS analysis, *Bunium persicum*, bioactive compounds, principal component analysis, Pearson correlation analysis, hierarchical clustering, network analysis

## Abstract

The present investigation was performed to evaluate the variability of the essential oil composition present in the seed extract of Kala zeera (*Bunium persicum* Bioss.) obtained from different geographical zones of Northwestern-Himalayan using Gas Chromatography-Mass Spectrum (GC-MS). The results of the GC-MS analysis revealed significant differences in the essential oil content. Significant variability was observed in the chemical constituents of the essential oils mainly for p-cymene, D-limonene, Gamma-terpinene, Cumic aldehyde and 1, 4-p-menthadien-7-al. Among these compounds, the highest average percentage across the locations was observed for gamma-terpinene (32.08%) which was followed by cumic aldehyde (25.07%), and 1, 4-p-menthadien-7-al (15.45%). Principal component analysis (PCA) also grouped the 4 highly significant compounds i.e., p-Cymene, Gamma-Terpinene, Cumic aldehyde, and 1,4-p-Menthadien-7-al into same cluster which are mainly distributed in Shalimar Kalazeera-1, and Atholi Kishtwar zones. The highest value of gamma-terpinene was recorded in Atholi accession (40.66%). However, among climatic zones Zabarwan Srinagar and Shalimar Kalazeera-1 was found to have highly positive significant correlation (0.99). The cophenetic correlation coefficient (c) was found to be 0.8334 during hierarchical clustering for 12 essential oil compounds showing that our results are highly correlated. Network analysis also showed the overlapping pattern and similar interaction between the 12 compounds as shown by hierarchical clustering analysis. From the results, it could be concluded that existence of variability among the various bioactive compounds of *B. persicum* which are probably to be incorporated to the potential list of drugs and may serve as good genetic source for various modern breeding programs.

## 1. Introduction

Herbs and spices have been used as preservatives to improve the flavor and organoleptic characteristics of food since ancient times [1]. In recent years, bio-active compounds extracted from different species of medicinal and edible plants influenced a great deal of scientific attention due to their capability to act as a source of natural agents to enhance the shelf life and safety of foods and natural biologically active compounds [2]. Among them, essential oils are one of the important bioactive molecules that are produced by aromatic and medicinal plants and have a wide range of applications in the food, fragrance, cosmetic, and pharmaceutical industries [3]. They have been widely used as a food preservative for grains, cereals, pulses, vegetables and fruits [3,4,5]. Essential oils are concentrated liquids comprised of complex combinations of volatile molecules that can be collected from a variety of plant organs [6].

The odor and flavor of essential oils found in many different plants, particularly aromatic plants, varies according to the types and quantities of elements present in oils. Generally, essential oils contain terpenes (monoterpenes and sesquiterpenes), aromatic compounds (aldehyde, phenol, alcohol, methoxy derivative), and terpenoids (isoprenoids) [7,8]. Furthermore, the amount of essential oil extracted from different plants varies, which influences the price of essential oil. Because of the growing interest in natural additives, essential oils from various plants have become more commonly used, particularly in combination with other preservations under the notion of “hurdle technology”. As a result, essential oils can be used as an alternative addition or as a processing aid in green technology. In addition, due to user concerns about synthetic preservatives, there is now a greater emphasis on natural preservatives such as essential oils. However, various factors, including climatic, geographic, and growth stages of harvested plants, can have a significant impact on essential oil yield, content, and biological qualities [9]. Thus, research on essential oil chemical variability in response to environmental and geographical conditions may provide insight into what causes chemical polymorphism. Furthermore, deciphering the chemical composition of essential oils is a critical quality requirement for their marketing and contributes to their value.

In this work, we systematically examined the variability in the chemical composition of essential oils of *B. persicum* Bioss commonly known as Kala zeera. It is one of the most important spice crops belonging to Umbellifereae (now called as Apiaceae) family that includes 423 genera and 3000 species of predominantly aromatic herbs distributed throughout the world especially in the northern hemisphere [10]. The important characteristic feature of the Apiaceae family is its inflorescence “umbel,” which means “sunshade” as it is a flat-topped or convex-shaped cluster of flowers in which all the pedicles arise from the same apex [11]. Kala zeera is a tuberous and perennial herb that is either compact or spreading, moderately to vigorously branched and varies from dwarf (30 cm) to tall (80 cm). The leaves are filiform, freely pinnate (2–3) and finely dissected. The flowers are small and white with sepals, petals and stamens arranged in symmetrical order [12]. Kala zeera might is believed to be native of Central Asia and Northern India. The range of kala zeera extends though most in alpine and sub-alpine habitats of north western himalayas at an altitude of 1800–3500 m amsl. It occurs abundantly in forests, grassy slopes, and comparatively in low alpine pastoral lands in Jammu, Himachal Pradesh, Uttaranchal and Kashmir regions. In Jammu and Kashmir, Kala zeera is grown in Karewas regions of Budgam and Srinagar, hilly regions of Tangdar, Machill, Gurez, Pulwama, Padder and Karnah [13]. Mushtaq and Modi, [14], analyzed the morphological characteristics of different ecotypes of Kala zeera.

*Bunium persicum* is a well-known spice around the world for its numerous biochemical and therapeutic qualities because to its high concentration of bioactive components such as aldehydes and terpenes. Presence of various bio-active compounds indicates the medicinal importance since it shows several therapeutic effects on urinary tract and digestive problems and is also used for the treatment of diarrhea, dyspepsia, fever, flatulence, stomach ache, hemorrhoids, and hiccoughs and as an antihistaminic [15,16]. It is a high value herbaceous spice widely used for culinary, flowering, perfumery and carminative purposes [16]. The plant is also used for culinary purposes and for flavoring foods and beverages [17]. The essential oil of *B. persicum* is reported to suppress the initial stage of inflammatory response as it exhibit antibacterial [18,19], antioxidative [20], and antifungal properties [21]. The ripe fruits act as valuable spice for flavoring foods and exhibits lactagogue, expectorant, carminative, diuretic and antispasmodic properties [22]. *B. persicum* seeds are rich source of essential oil (7%), with abundant amount of monoterpene aldehydes as P-mentha-1, 4-dien-7-al (28.98%), gamma-terpinene (25.72%), beta-pinene (15.62%) and cumin aldehyde (11.71%) [15,16,23]. The seeds of *B. persicum* possess expectorant, stimulant, antispasmodic and diuretic properties and are reported to have medicinal importance as commonly used for curing numerous diseases as dyspepsia, diarrhea, fever and stomach problems [24,25,26,27].

Essential oils are complex natural mixtures that are composed of 20–60 chemical components at quite different concentrations. Chemically, these are the mixture of terpenes and terpenoids that are usually present in the form of minute droplets between cells and synthesized in the cytoplasm [27]. These are aromatic, volatile, highly lipophilic components that are insoluble in water but readily soluble in organic solvents [28]. The essential oils have remarkable antioxidative, antifungal and antibacterial properties [19,20,22]. The essential oil composition is affected by various factors such as genetic composition, geographical distribution, drying techniques, distillation time, distillation method [29,30] climatic and seasonal conditions, [15,31] harvesting season, harvest period and time [32], and plant growth regulators [33].

The essential oils and the herbal extracts from various species of edible and medicinal plants have attracted a great deal of scientific interest due to their potential as a source of natural agents to increase the safety and shelf life of foods and of natural biologically active compounds [34]. Since, extraction of essential oils is a challenging aspect for researchers, unsuitable and improper method of extraction can alter and destruct the biological activity of chemical components present in essential oils. Conventionally available extraction methods as steam distillation, hydro-distillation, cold expression is time consuming and complicated. Nowadays, effective and low-cost extraction methods are adopted that are promising alternatives for conventional methods as supercritical fluid extraction, accelerated solvent extraction, microwave hydrodiffusion. However, modern separation technologies as ultrafiltration, column chromatography, high performance liquid chromatography (HPLC) proves highly efficient for the extraction of chemical compounds.

In phytochemistry gas chromatography mass spectrometry (GC-MS) has proven to be a valuable approach for identifying bioactive components, long chain hydrocarbons, alcohols, esters, acids, alkaloids, and steroids. GC-MS is a key technological platform and got firmly established for profiling of secondary metabolites in both plant and non-plant species [35,36,37].

In spite of prized herbs having many therapeutic uses, the information on phytochemical evaluation among the geographically isolated populations is severely lacking. Moreover, phytochemical evaluation is necessary to identify genotypes that possess high concentration specific active principles, which can be included in the breeding program for further improvements of phytochemical traits [6]. Therefore, it is necessary to identify and select different genotypes for different phytochemicals for cultivation and production for the benefit of farming community and industrial purposes. Thus, the aim of this study is to investigate the essential oil content and to study variability among the compounds and thus elucidating potential sources of ethnic medicinal plants among ecotypes of *B. persicum* collected from different regions which are likely to be added to the potential list of drugs.

## 2. Results and Discussion

### 2.1. Isolation and Chemical Characterization of Essential Oils from B. persicum Using GC-MS Analysis

**Figure 1:**
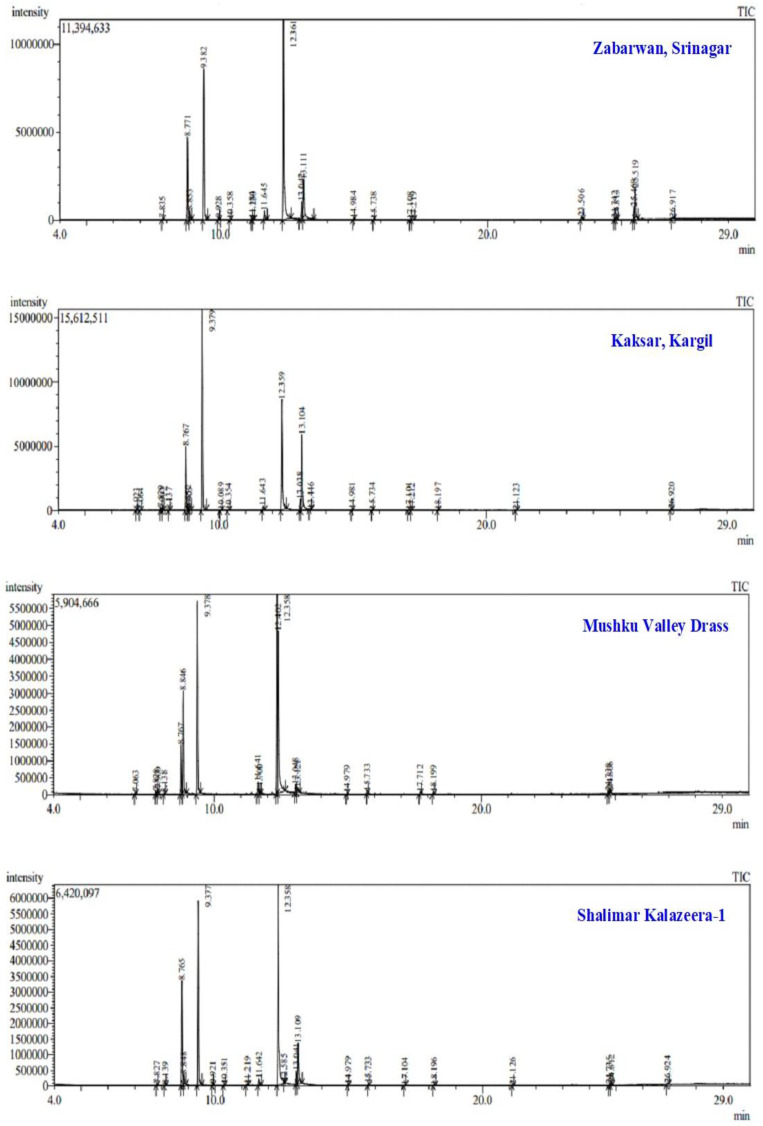

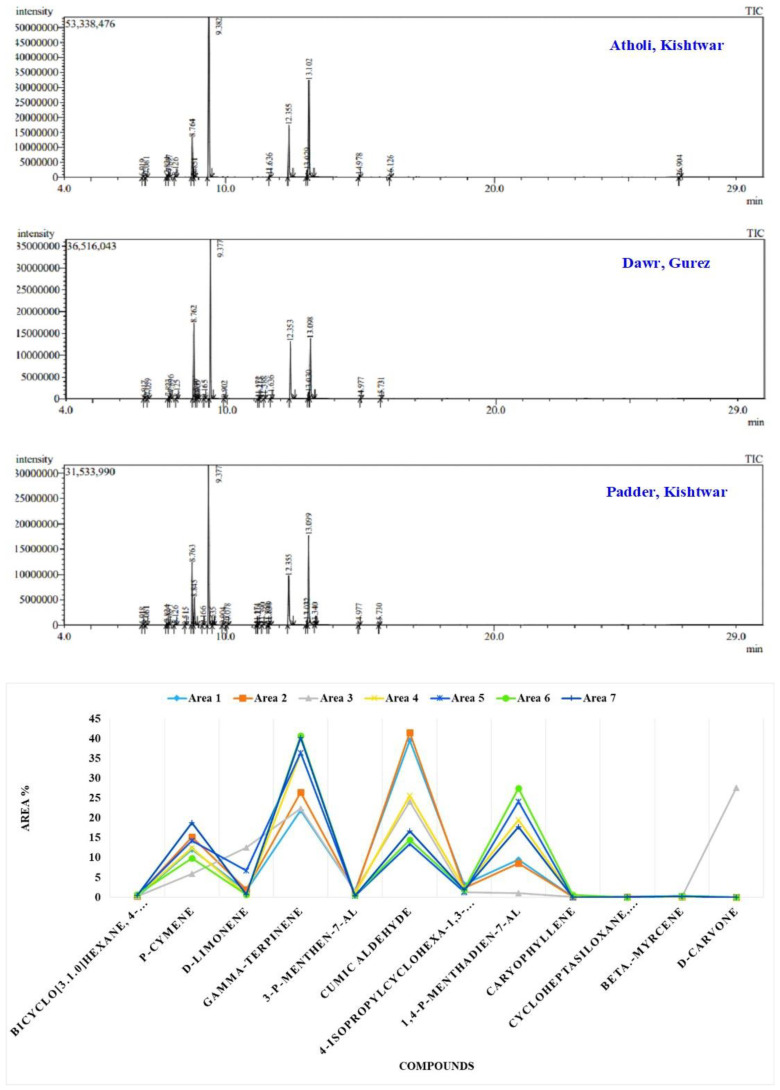
Figure showing the chemical composition and variability of essential oils (*X*-axis) in *B. persicum* collected from seven different geographical locations as area percent (*Y*-axis).

Forty-two compounds of the essential oils were isolated and further characterized with GC–MS analysis. GC-MS analysis of each sample was carried out on Agilent 7890 AGC, furnished with an HP-5 MS capillary column (30 m × 0.250 mm, 0.25 mm) and an HP 5975 C mass selective detector was employed for the analysis. Helium was used as the carrier gas with flow rate of 1.00 mL/min. Column temperature was initially programmed at 50 °C held for 3 min then increased to 150 °C at the rate of 3 °C/min and finally increased to 250 °C at the rate of 10 °C/min. Sample was diluted in hexane 1:100 *v*/*v* of which 1.0 µL was injected automatically in split less mode. The ionization energy was 70 eV and electron emission 100 µA. The compounds represent 97.0 to 98.0 per cent of the total essential oil composition of the *B. persicum* seeds. High variability in the chemical composition of the essential oils was observed mainly for p-Cymene, D-Limonene, Gamma-Terpinene, Cumic aldehyde, and 1, 4-p-Menthadien-7-al (Figure 1). The detailed list of essential oil composition in kala zeera from seven different locations including retention time, area%, similarity, and base *m*/*z* has been shown in supplementary Tables S1–S7.

Among these compounds, the highest average percentage across the locations was observed for Gamma-Terpinene (32.08%) which was followed by Cumic aldehyde (25.07%) and 1,4-p-Menthadien-7-al (15.45%). Highest Gamma-Terpinene was recorded in Atholi accession (40.66%) which was closely followed by Padder Valley accession (40.22%) while lowest percentage was observed in Zabarwan accession (21.92%). Similarly, the highest and lowest percentage of Cumic aldehyde was observed in Shalimar Kalazeera-1 (41.54%) and Padder Valley accession (13.44%), respectively (Table 1). The total content of these 3 compounds accounted for more than half of the total amount of the oil composition. One compound namely D-Carvone was observed only in Kaksar Kargil accession showing the highest content (27.6%) in the Mushku Valley and second highest among all other essential oil content analyzed (Table 1).

The present results are supported by the findings of Pourmortazavi et al. [38] who analyzed chemical constituents using GC-MS in *B. persicum* extract and identified a-methyl benzenemethanol (26%), c-terpinene (38%) and cuminaldehyde (11%) as the major compounds among 16 compounds. Further, Shahsavari et al., [20] also analyzed essential oils composition of the seed of *B. persicum* by employing GC-MS technique and analyzed caryophyllene (27.81%), ϒ-terpene (15.19%), cuminyl acetate (14.67%) as the major components. Azizi et al. [39] identified c-terpinene as the major component, which was associated with cuminaldehyde and c-terpinen-7-al among two wild population of *B. persicum*. Foroumadi et al., [16] identified 25 components by using GC-MS and observed cuminaldehyde (27.0%), ϒ-terpene (25.8%), P-cymene (12.14%), cuminyl alcohol (6.0%) and limonene (5.1%) as the major components of essential oil of *B. persicum*.

The concentration range of main components of essential oils in kala zeera collected from different locations of Kashmir valley were Cumin aldehyde (13.44–41.54%), Gamma-Terpinene (21.92–40.66%), 1,4-p-Menthadien-7-al (1.12–27.51%), 4-Isopropylcyclohexa-1,3-dienecarbaldehyde (1.33–3.25%), 3-p-Menthen-7-al (0.33–1.64%) (Table 1). Present results are in accordance with the findings of Thappa et al. (1991) who revealed that essential oil of seed extract of cultivated plants is mainly composed of cuminaldehyde (27.3–34.1%), a-terpinen-7-al and c-terpinen-7-al (29.6–36.8%), while as c-terpinene (25.6–42.9%) and p-cymene (24.0–27.8%) was observed as the main components and less aldehyde value in wild collected seeds. Further, Mazidi et al., [40] revealed that ϒ-terpene (28.16–31.13% *w*/*w*), cuminaldehyde (24.85–29.20%), p-cymene (14.67–16.50%) and limonene (16.13–8.28%) were their main constituents of essential oils by employing GC-MStechnique with a similar composition both after conventional hydrodistillation (HD) and microwave-assisted hydrodistillation (MAHD) extraction.

Omidbaigi and Arvin, [41] revealed that growing locations have a major impact on compositions and content of essential oil of *B. persicum* fruits and observed huge variation in cuminaldehyde concentration among the seeds of two Iranian sites. Jahansooz et al. [42] also revealed geographical differentiation affect the oil composition as indicated by the concentration of c-terpinene (39.7–41.9%), a-terpinen-7-al (37.2%) and cuminaldehyde (37.1%), among three Iranian, one Pakistan and one Indian population, respectively. The present investigation is further justified by the studies of various researcher who observed the essential oil profiles of aromatic plants are remarkably affected by different factors. They revealed that chemical composition of essential oils was dependent on various factors such as genetic composition [43], environmental conditions such as climate, altitude [44,45] and plant habitat [46].

According to the present result and previous investigation of various research on the composition of *B. persicum* fruit oil, it can be depicted that occurrence of high variability in the chemical composition of the essential oils that confirms the existence of chemotypes. Differences may be either due to variation at genetic level, fluctuation in environmental conditions (soil, climate), geographical distribution, harvesting techniques and period, and drying techniques. Present results depicted that quantity and quality of chemical constituents of essential oil was greatly affected by environmental factors. Moreover, the production and chemical composition of plant essential oils also gets affected by external applications of plant growth regulators in the plant [33].

### 2.2. Principal Component Analysis between 12 Different Compounds and Seven Climate Zones of North Western Himalayas

Principal component analysis (PCA) based on score values between 12 different compounds (including Bicyclo[3.1.0]hexane, 4-methylene-1-(1-methylethyl)-, p-Cymene, D-Limonene, Gamma-Terpinene, 3-p-Menthen-7-al, Cumic aldehyde, 4-Isopropylcyclohexa-1,3-dienecarbaldehyde, 1,4-p-Menthadien-7-al, Caryophyllene, Cycloheptasiloxane, tetradecamethyl-, beta.-Myrcene, and D-Carvone) and seven climatic zones (Zabarwan Srinagar, Shalimar Kalazeera-1, Mushku Valley Drass, Kaksar Kargil, Padder Valley Kishtwar, Atholi Kishtwar, and Dawr Gurez) of North Western Himalayas has been shown in Table 2.

**Figure 2:**
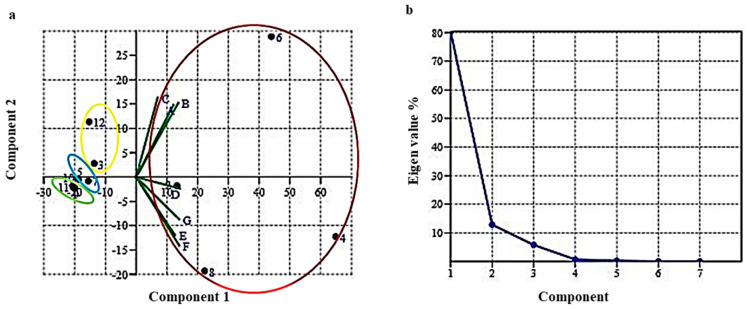
Graphical representation of principal component analysis (PCA) showing the matrix of two variables (**a**) Scatter plot between *X*-axis (PC1) and *Y*-axis (PC2) showing the distribution of 12 different compound and seven climate zones of North Western Himalayas. Where 12 different componds were clustered into four circles and denoted as red, yellow, blue and green circles and (**b**) Scree plot showing eigen values at *Y*-axis and number of factors/components at *X*-axis.

PCA has grouped the 12 essential oil compounds of kala zeera into 4 clusters based on scatter plot graph (Figure 2a). Cluster 1 (in red circle) has 4 compounds i.e., p-Cymene, Gamma-Terpinene, Cumic aldehyde, and 1,4-p-Menthadien-7-al followed by Cluster 2 (in yellow circle) containing total 2 compounds i.e., D-Carvone and D-Limonene; Cluster 3 (in blue circle) containing total 2 compounds i.e., 3-p-Menthen-7-al and 4-Isopropylcyclohexa-1,3-dienecarbaldehyde; and Cluster 4 (in green circle) containing total 4 compounds i.e., Bicyclo[3.1.0]hexane 4-methylene-1-(1-methylethyl)-, Caryophyllene, Cycloheptasiloxane tetradecamethyl-, and beta.-Myrcene, respectively. In addition, the seven climatic zones of North Western Himalayas were found in two groups as Group I (including the regions Zabarwan Srinagar, Shalimar Kalazeera-1, and Mushku Valley Drass), and Group 2 (including the regions Kaksar Kargil, Padder Valley Kishtwar, Atholi Kishtwar, and Dawr Gurez) as shown in Figure 2a.

The PC1 have shown the highest positive scores for the essential oil compounds viz., Gamma-Terpinene (64.874), Cumic aldehyde (43.963), and 3-p-Menthen-7-al (22.24) as shown in Table 2. In addition, the more positive loadings indicating that a variable and a PC are positively correlated were observed in PC3 for the essential oil compound Gamma-Terpinene (0.849), followed by PC5 and PC6 for D-Limonene (0.842) and 4-Isopropylcyclohexa-1,3-dienecarbaldehyde (0.696), respectively, as shown in Table 3.

Panwar, [12] reported that the main component of essential oil present in kala zeera are monoterpene aldehyde (4–15%) in the cultivated accessions which includes cumin aldehyde, pmentha-1, 3-dien-7-al and p-mentha-1,4-dien-7-a. We have also found cumin aldehyde and p-mentha-1,4-dien-7-a as the highest expressed essential oils in the Shalimar Kalazeera-1 and Atholi Kishtwar Northwestern Himalayas climatic zones, respectively. Similarly, we have also found that the highest expression of major volatiles that are majorly present in wild cultivars viz., γ-terpinene and p-cymene in Dawr Gurez and Atholi Kishtwar zones that revealed the flavor of kala zeera into same cluster.

However, the clustering of PCA between component 1 and component 2 divided the seven climatic zones based on the highest percent of eigen values (Euclidean similarity index (index) (80.39% to 0.001%), has been shown in scree plot (Figure 2b). PCA based on variance-covariance matrix demonstrated that maximum variance was observed in PC1 i.e., Zabarwan Srinagar (80.39%) followed by PC2 Shalimar Kalazeera-1 (12.86%) and minimum in PC6 i.e., Atholi Kishtwar (0.008%) followed by PC7 i.e., Dawr Gurez (0.001%) as shown in Table 4 and Figure 2b.

### 2.3. Correlation, Cluster and Network Analysis between 12 Different Compounds and Seven Climate Zones of North Western Himalayas

**Figure 3:**
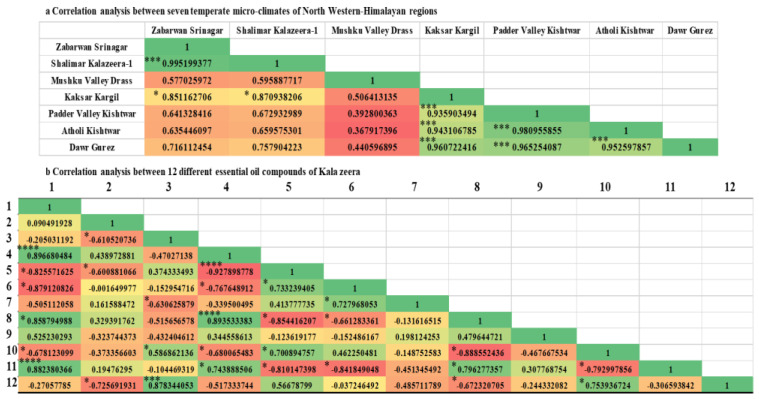
Pearson correlation analysis showing the strength of linear relationship between two components. (**a**) Correlation analysis between seven temperate micro-climates of Northwestern-Himalayan region, and (**b**) Correlation analysis between 12 different essential oil compounds of Kala zeera. Where 1: Bicyclo[3.1.0]hexane, 4-methylene-1-(1-methylethyl)-; 2: p-Cymene; 3: D-Limonene; 4: Gamma-Terpinene; 5: 3-p-Menthen-7-al; 6: Cumic aldehyde; 7: 4-Isopropylcyclohexa-1,3-dienecarbaldehyde; 8: 1,4-p-Menthadien-7-al; 9: Caryophyllene; 10: Cycloheptasiloxane, tetradecamethyl-; 11: beta.-Myrcene; and 12: D-Carvone. The result is significant at *p* < 0.05, where flag stars de-notes the level of significance. *p*-value < 0.05 is flagged with one star (*), *p*-value < 0.001 is flagged with 3 stars (***) and *p*-value < 0.0001 is flagged with 4 stars (****).

Pearson correlation analysis at (*p* value 0.05) between seven climate zones of Northwestern Himalayas demonstrated significant variation a shown in Figure 3a. Zabarwan Srinagar and Shalimar Kalazeera-1 was found to have highly positive significant correlation (0.99) followed by Padder Valley Kishtwar and Atholi Kishtwar (0.98), Padder Valley Kishtwar and Dawr Gurez (0.97), Kaksar Kargil and Dawr Gurez (0.96), Atholi Kishtwar and Dawr Gurez (0.95), respectively. Among 12 different oil compounds of Kalazeera Bicyclo[3.1.0]hexane, 4-methylene-1-(1-methylethyl)- and Gamma-Terpinene (0.895) followed by 1,4-p-Menthadien-7-al and Gamma-Terpinene (0.893), Bicyclo[3.1.0]hexane, 4-methylene-1-(1-methylethyl)- and beta.-Myrcene (0.88), D-Limonene and D-Carvone (0.87), and Bicyclo[3.1.0]hexane, 4-methylene-1-(1-methylethyl)- and 1,4-p-Menthadien-7-al (0.86) was found to have high positive correlation among all the compounds studied (Figure 3b).

**Figure 4:**
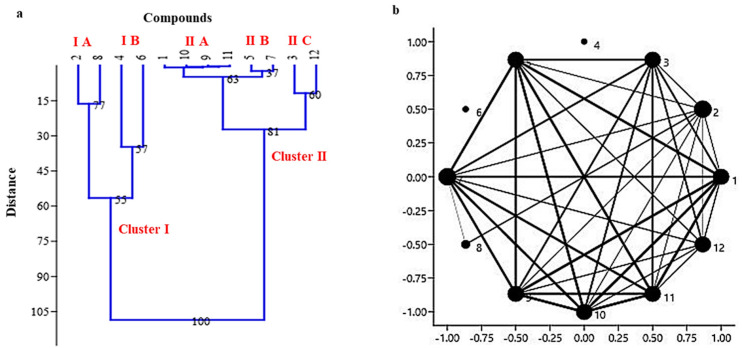
(**a**) Dendrogram showing the hierarchical clustering to interpret the distribution of 12 different essential oil compounds of *B. persicum*, and (**b**) Network clustering to extract the densely connected but relatively isolated subnetworks component of 12 different essential oil compounds of *B. persicum*.

Hierarchical clustering was analyzed using euliciden similarity index and ward’s method algorithm for 12 oil compounds at seven different climatic zones. The cophenetic correlation coefficient (c) was found to be 0.8334 at bootstrap value N = 1000 showing that our results are highly correlated. This study classified the compounds into two main clusters I and II as shown in Figure 4a. Further, Cluster I was divided into two sub-clusters (IA and IB) while Cluster II was divided into three sub-clusters (IIA–IIC). While selecting the highly induced compound 12 (D-Carvone as 27.6%) in Mushku Valley, Drass as an outgroup, the next four average high content of oil compounds 4, 6, 2, and 8 (namely p-Gamma-Terpinene as 32.08%, Cumic aldehyde as 25.07%, 1,4-p-Menthadien-7-al as 15.45%, and Cymene as 12.66%) were found be present in the same cluster I (or sub cluster IA and IB) showing highest value in the individual zones of Dawr Gurez, Atholi Kishtwar, Shalimar Kalazeera-1, and Atholi Kishtwar, respectively (Table 1). However, the lowest value of the oil compounds was found to be present in a cluster II which includes II B containing 3-p-Menthen-7-al (0.94%), and 4-Isopropylcyclohexa-1,3-dienecarbaldehyde (2.06%) followed by Cluster II A containing Bicyclo[3.1.0]hexane 4-methylene-1-(1-methylethyl)- (0.41%), Caryophyllene (0.2%), Cycloheptasiloxane tetradecamethyl- (0.14%), beta.-Myrcene (0.23%), respectively (Table 1). The highest zonal distribution of these compounds were found to be present in Mushku Valley Drass, Zabarwan Srinagar, Atholi Kishtwar, Atholi Kishtwar, Mushku Valley Drass, and Atholi Kishtwar, respectively (Table 1). However, the oil compounds D-Limonene (12.59%) and D-Carvone (27.6%) was found to be present in same sub cluster II C showing the highest zonal distribution at Mushku Valley, Drass. Overall, most of the oil compounds present in Kala zeera were present in Atholi Kishtwar (includes 5 oil compounds) followed by Mushku Valley Drass (includes 4 oil compounds) (Table 1).

Network analysis has also shown a similar interaction between the 12 compounds as shown by hierarchical clustering analysis. The oil compounds showing highest value (namely 4, 6, and 8) were found to be distributed separately in the network analysis graph, followed by the highest interaction between the compounds (namely 5, 7, 1, 9, 10, and 11) present in cluster IIA and IIB (Figure 4b).

Investigation on the phytochemical evaluation on the basis of GC-MS analysis revealed significant variability among the chemical constituents of the essential oils mainly for p-cymene, D-limonene, Gamma-terpinene, Cumic aldehyde and 1, 4-p-menthadien-7-al. Considerable percentage of D-Carvone was detected only in two population i.e., Kaksar Kargil and Mushku Valley of accession showing the highest percentage although it was completely absent in rest of populations. The highest Gamma-Terpinene was recorded in Atholi accession (40.66%) and Padder Valley accession (40.22%) while lowest percentage was observed in Zabarwan accession (21.92%). Similarly, the highest and lowest percentage of Cumic aldehyde was observed in Shalimar Kalazeera-1 (41.54%) and Padder Valley accession (13.44%), respectively. However, among climatic zones the genotypes Atholi Kishtwar and Mushku Valley Drass accessions possess the most essential oil yield so they were supposed to large cultivation and can be used for desirable hybridization. According to the available results, it is better at the time of selection, these samples should be used for mass cultivation with high essential oil content. Since the prevailing climatic regimes and adaptive behavior of plant towards these regimes can alter the genetic variation that can have direct or indirect impact play on the biological activity and composition of an active principle [16,47].

## 3. Materials and Methods

### 3.1. Collection of Plant Material

In order to find the variability among different ecotypes of *B. persicum* seven locations as Zabarwan Srinagar, Shalimar Kalazeera-1, Mushku Valley Drass, Kaksar Kargil, Padder Valley Kishtwar, Atholi Kishtwar and Dawr Gurez were selected (Table 5). To minimize any loss to fruits hand threshing was done which was further cleaned through round holes sieve (2 mm diameter). Manual separation of fruits from similar size particles, empty or half-filled seed were also done. Later on, drying of samples was done in a shade, cool, dry ventilated place (24 ± 1 °C) which were stored at 4–6 °C prior to chemical analysis.

### 3.2. Essential Oil Isolation

The air-dried 50 g seeds of *B. persicum* were used to determine the oil content (*v*/*w*%) by hydro-distillation extraction for 3 h in clevenger-type apparatus as recommended in standard protocol described in the European Pharmacopoeia. The resulting essential oil samples were collected which was further dried over sodium sulfate (Na_2_SO_4_) and stored in sealed amber vials in refrigerator at +4 °C prior to analysis of components. Thereafter, the obtained oil samples were subjected to GC-MS analysis.

### 3.3. GC-MS Analysis Conditions

Essential oil components were analyzed at Quality Control Laboratory SKUAST-Kashmir. Agilent 7890 AGC (Santa Clara, CA, USA), furnished with an HP-5 MS capillary column (30 m × 0.250 mm, 0.25 mm) and an HP 5975 C mass selective detector was employed for the analysis of essential oil. For GC-MS detection electron ionization energy of 70 eV was kept and helium at a flow rate of 1 mL/min was used as a carrier gas. Temperatures of the injector and MS transfer line were set in the same order at 220 and 290 °C, respectively. The column temperature program comprises of three temperature levels. Initially, the temperature was maintained at 50 °C for the 3 min, followed by 3 °C/min rate up to 150 °C. Finally, the temperature rose to 250 °C with 10 °C/min in 10 min. Dilution of samples was done (1/100 in hexane, *v*/*v*) of 1.0 µL which were injected automatically in split less mode. The ionization energy was 70 eV and electron emission 100 µA. The identification of different components was done by comparing their relative retention time and mass spectra with the predetermined standards, NIST05a, Wiley library data of GC-MS system and further with the authentic descriptions [48]. The means of essential oil contents of harvests were subjected to standard deviation using Microsoft excel (Redmond, WA, USA). The experiment for determining variability’s in essential oil content was laid in a completely randomized block design with 3 replicates.

### 3.4. Statistical Analysis

Multivariate analysis based on PCA (Principal component analysis) as well as cluster analysis based on dendrogram was performed in seven North Western-Himalayan regions to assess the variability in chemical composition of essential oils of Kala zeera. Further, Pearson correlation analysis and network analysis were performed to estimate the significant correlation within and between the seven different temperate regions and twelve metabolic compounds present in oils of Kala zeera. All statistical analysis was performed using Microsoft excel and Past (PAleontological STatistics) 4.03 software (Norway, Europe).

## 4. Conclusions

The present study aimed at identifying the variability in the composition of essential oil in different accessions of *B. persicum* isolated from different geographic locations and altitudes. According to the present investigation, significant diversity in the chemical compositions of the essential oil contents was observed among different accessions of *B. persicum*. The differences in the chemical composition and oil content of different populations may be due to genetic variability and thus may serve as a good genetic source for various modern breeding programs. In the future, these essential oils can be exploited as natural additives in foods as well as their therapeutic potential. However, metabolic and biotechnological engineering can be used to comprehend the biosynthetic pathways of these bioactive compounds and to develop superior *B. persicum* cultivars rich in essential oil with more palatable flavor that will be of great value in various food and therapeutic industries.

## Figures and Tables

**Table 1:** Essential oil composition in different accessions of *B. persicum* collected from different climates of Northwestern Himalayas.

Compound	Area (%)							Mean (µ)	SD (σ)
	1	2	3	4	5	6	7		
Bicyclo[3.1.0]hexane, 4-methylene-1-(1-methylethyl)-	0.17	0.24	0.29	0.40	0.53	0.71	0.50	0.41	0.19
p-Cymene	12.11	15.18	5.98	12.44	14.27	9.88	18.76	12.66	4.06
D-Limonene	1.64	1.95	12.59	0.71	6.78	0.71	0.66	3.58	4.52
Gamma-Terpinene	21.92	26.51	22.45	36.36	36.42	40.66	40.22	32.08	8.21
3-p-Menthen-7-al	1.59	1.13	1.64	0.94	0.33	0.56	0.36	0.94	0.55
Cumic aldehyde	39.55	41.54	24.10	25.64	13.44	14.53	16.72	25.07	11.53
4-Isopropylcyclohexa-1,3-dienecarbaldehyde	3.25	2.32	1.33	2.33	1.38	1.96	1.83	2.06	0.66
1,4-p-Menthadien-7-al	9.57	8.56	1.12	19.47	24.15	27.51	17.78	15.45	9.40
Caryophyllene	0.18	0.17	0.09	0.22	0.04	0.61	0.07	0.20	0.19
Cycloheptasiloxane, tetradecamethyl-	0.09	0.19	0.28	0.14	0.04	-	0.09	0.14	0.09
beta.-Myrcene	0.16	0.11	0.15	0.14	0.38	0.39	0.31	0.23	0.12
D-Carvone	-	-	27.60	-	-	-	-	27.60	-

where Area 1: Zabarwan, Srinagar; Area 2: Shalimar Kalazeera-1; Area 3: Mushku Valley Drass; Area 4: Kaksar, Kargil; Area 5: Padder Valley, Kishtwar; Area 6: Atholi, Kishtwar: Area 7: Dawr, Gurez.

**Table 2:** Principal component analysis (PCA) scores showing correlation coefficients for the variables loaded between the rows for 12 different compound variables and the columns for seven climate zones of North Western Himalayas variables.

Compound	PC 1	PC 2	PC 3	PC 4	PC 5	PC 6	PC 7
Bicyclo[3.1.0]hexane, 4-methylene-1-(1-methylethyl)-	−19.877	−2.3037	−3.0839	−0.1287	−0.7254	−0.2367	0.08317
p-Cymene	13.266	−1.7894	−4.4934	−6.6893	1.5689	0.38245	0.10253
D-Limonene	−13.574	2.7936	7.0385	0.40952	4.4759	−0.3107	−0.1015
Gamma-Terpinene	64.874	−12.262	8.0665	−1.6293	−1.5443	−0.3239	−0.0913
3-p-Menthen-7-al	−18.691	−0.4942	−2.8329	0.20041	−0.7894	0.05656	−0.1665
Cumic aldehyde	43.963	28.866	−8.4323	2.0817	0.0748	−0.0287	0.02121
4-Isopropylcyclohexa- 1,3-dienecarbaldehyde	−15.503	−0.8088	−3.8088	0.22301	−0.9262	0.48871	−0.219
1,4-p-Menthadien-7-al	22.24	−19.324	−2.84	5.2228	1.3014	0.34354	0.10481
Caryophyllene	−20.422	−2.0897	−3.3217	0.1183	−0.9407	−0.2357	0.1578
Cycloheptasiloxane, tetradecamethyl-	−20.686	−1.7734	−3.194	−0.2515	−0.7236	−0.3012	−0.0644
beta.-Myrcene	−20.34	−2.1809	−3.2041	−0.1842	−0.6225	−0.1488	0.07826
D-Carvone	−15.252	11.367	20.106	0.6271	−1.149	0.31443	0.0949

where seven climate zones of Northwestern Himalayas denoted as principal component (PC) 1: Zabarwan Srinagar; PC 2: Shalimar Kalazeera-1; PC 3: Mushku Valley Drass; PC 4: Kaksar Kargil; PC 5: Padder Valley Kishtwar; PC 6: Atholi Kishtwar; and PC 7: Dawr Gurez.

**Table 3:** Principal component analysis (PCA) loadings between seven climate zones of Northwestern Himalayas.

	PC 1	PC 2	PC 3	PC 4	PC 5	PC 6	PC 7
Zabarwan, Srinagar	0.36103	0.43949	−0.36015	0.21627	0.10026	0.69623	−0.072439
Shalimar, Kalazeera-1	0.40537	0.45059	−0.33667	−0.15151	0.071625	−0.5932	0.37326
Mushku Valley, Drass	0.20698	0.48385	0.84939	0.030137	−0.015923	0.019998	0.0043217
Kaksar, Kargil	0.42256	−0.070291	−0.063804	0.16946	−0.30868	−0.29656	−0.77491
Padder Valley, Kishtwar	0.37377	−0.35323	0.12638	0.029115	0.84215	−0.044791	−0.086501
Atholi, Kishtwar	0.41537	−0.41703	0.10769	0.53065	−0.34112	0.039087	0.49243
Dawr, Gurez	0.41437	−0.25708	0.061978	−0.78626	−0.2524	0.26738	0.070434

where PC1: Zabarwan, Srinagar; PC2: Shalimar, Kalazeera-1; PC3: Mushku Valley, Drass; PC4: Kaksar, Kargil; PC5: Padder Valley, Kishtwar; PC6: Atholi, Kishtwar; PC7: Dawr, Gurez.

**Table 4:** Eigen value and percent (%) variance of seven principal component (PC) that includes different climates of Northwestern Himalayas.

PC	Eigen Value	% Variance
Zabarwan, Srinagar	861.114	80.393
Shalimar, Kalazeera-1	137.792	12.864
Mushku Valley, Drass	61.9786	5.7863
Kaksar, Kargil	7.25369	0.6772
Padder Valley, Kishtwar	2.8821	0.26907
Atholi, Kishtwar	0.0938095	0.008758
Dawr, Gurez	0.0152141	0.0014204

**Table 5:** Details of collection sites of Kala zeera ecotypes.

S No	Ecotypes	Altitude (mts)	Coordinates (Degree)
1	Zabarwan Srinagar	1587	34.14 N, 74.96 E
2	Shalimar Kalazeera-1	1587	34.08 N 74.83 E
3	Mushku Valley Drass	3722	34.19 N, 75.33 E
4	Kaksar Kargil	2773	35.61 N, 75.81 E
5	Padder Valley Kishtwar	1640	33.15 N, 76.09 E
6	Atholi Kishtwar	1640	33.15 N, 76.09 E
7	Dawr Gurez	2580	34.63 N, 74.83 E

## Data Availability

Not applicable.

## Supplementary Materials

The following supporting information can be downloaded at: https://www.mdpi.com/article/10.3390/molecules28052404/s1, Table S1: Essential oil composition in Kala zeera (Zabarwan Srinagar collection); Table S2: Essential oil composition in Kala zeera (Shalimar Kalazeera-1collection); Table S3: Essential oil composition in Kala zeera (Mushku Valley Drass collection); Table S4: Essential oil composition in Kala zeera (Kaksar Kargil collection); Table S5: Essential oil composition in Kala zeera (Padder Valley Kishtwar collection); Table S6: Essential oil composition in Kala zeera (Atholi Kishtwar collection); Table S7: Essential oil composition in Kala zeera (Dawr Gurez collection).
