# Supplementary material for: Unraveling the Variability of Essential Oil Composition in Different Accessions of Bunium persicum Collected from Different Temperate Micro-Climates
Source: Molecules. 2023 Mar 6;28(5):2404. doi: 10.3390/molecules28052404 (PMC10005284; doi:10.3390/molecules28052404)
Supplement: Supplementary file 1 [file molecules-28-02404-s001.zip › molecules-2229696-supplementary.pdf]

Table S1: Essential oil composition in Kala zeera (Zabarwan Srinagar collection)

**Compound**	**Retention Time**	**Area%**	**Similarity**	**Base m/z**
Bicyclo[3.1.0]hexane, 4-methylene-1-(1-methylethyl)-	7.835	0.17	92	93.10
p-Cymene	8.771	12.11	96	119.10
D-Limonene	8.853	1.64	95	68.05
.gamma.-Terpinene	9.382	21.92	97	93.05
L-Fenchone	9.928	0.18	94	81.05
Fenchol	10.358	0.12	92	81.10
(3E,5E)-2,6-Dimethylocta-3,5,7-trien-2-ol	11.184	0.20	86	67.10
Hotrienol	11.230	0.19	79	71.00
3-p-Menthen-7-al	11.645	1.59	94	79.05
Cumic aldehyde	12.361	39.55	96	133.05
4-Isopropylcyclohexa-1,3-dienecarbaldehyde	13.042	3.25	94	79.05
1,4-p-Menthadien-7-al	13.111	9.57	95	79.05
Caryophyllene	14.984	0.18	90	91.00
Cycloheptasiloxane, tetradecamethyl-	15.738	0.09	85	73.05
1H-Cycloprop[e]azulen-7-ol, decahydro-1,1,7-trimethyl-4-m	17.108	0.15	84	159.10
beta.-Myrcene	16.892	0.16	88	68.17
Caryophyllene oxide	17.219	0.14	83	79.00
Hexadecanoic acid, ethyl ester	23.506	0.41	92	88.05
9,12-Octadecadienoic acid (Z,Z)-, methyl ester	24.742	0.18	86	67.05
6-Octadecenoic acid, methyl ester, (Z)-	24.813	0.50	94	55.05
Linoleic acid ethyl ester	25.468	1.95	94	67.05
Ethyl Oleate	25.519	5.46	90	55.05
1-Bromo-3,7-dimethyl-2,6-octadiene	26.917	0.32	78	69.05

Table S2: Essential oil composition in Kala zeera (Shalimar Kalazeera-1collection)

Compound	Retention Time	Area%	Similarity	Base m/z
Bicyclo[3.1.0]hexane, 4-methylene-1-(1-methylethyl)-	7.827	0.24	91	93.05
.beta.-Myrcene	8.139	0.11	87	93.00
p-Cymene	8.765	15.18	96	119.1
D-Limonene	8.848	1.95	94	68.05
.gamma.-Terpinene	9.377	26.51	97	93.05
L-Fenchone	9.921	0.19	94	81.05
Fenchol	10.351	0.11	89	81.05
(3E,5E)-2,6-Dimethylocta-3,5,7-trien-2-ol	11.219	0.39	83	71.00
3-p-Menthen-7-al	11.642	1.13	94	81.05
Cumic aldehyde	12.358	41.54	96	133.05
Cyclopropane,-1-methyl-1-ethenyl-2-(2-furyl)-,	12.585	0.03	61	133.10
4-Isopropylcyclohexa-1,3-dienecarbaldehyde	13.041	2.32	93	79.05
1,4-p-Menthadien-7-al	13.109	8.56	95	79.05
Caryophyllene	14.979	0.17	90	91.10
Cycloheptasiloxane, tetradecamethyl-	15.733	0.19	84	73.05
(3R,4aS,8aS)-8a-Methyl-5-methylene-3-(prop-1-en-2-yl)-1,	17.104	0.12	78	159.15
Cyclooctasiloxane, hexadecamethyl-	18.196	0.17	85	73.00
Cyclononasiloxane, octadecamethyl-	21.126	0.08	74	72.95
Methyl 10-trans,12-cis-octadecadienoate	24.735	0.19	82	67.05
6-Octadecenoic acid, methyl ester, (Z)-	24.812	0.54	89	55.00
7,8-Dibromo-4,4,7-trimethyl-hexahydro-benzo[1,3]dioxin-2	26.924	0.26	68	69.05

Table S3: Essential oil composition in Kala zeera (Mushku Valley Drass collection)

**Compound**	**Retention Time**	**Area%**	**Similarity**	**Base m/z**
.alpha.-Pinene	7.063	0.18	93	93.05
Bicyclo[3.1.0]hexane, 4-methylene-1-(1-methylethyl)-	7.828	0.29	94	93.05
Bicyclo[3.1.1]heptane, 6,6-dimethyl-2-methylene-, (1S)-	7.900	0.54	96	93.05
.beta.-Myrcene	8.138	0.15	89	93.00
p-Cymene	8.767	5.98	96	119.10
D-Limonene	8.846	12.59	96	68.05
.gamma.-Terpinene	9.378	22.45	97	93.05
3-p-Menthen-7-al	11.641	1.64	95	79.00
Cyclohexanone, 2-methyl-5-(1-methylethenyl)-, trans-	11.700	0.23	82	67.05
Cumic aldehyde	12.358	24.1	96	133.05
D-Carvone	12.402	27.6	95	82.05
4-Isopropylcyclohexa-1,3-dienecarbaldehyde	13.048	1.33	93	79.05
1,4-p-Menthadien-7-al	13.121	1.12	92	79.05
Caryophyllene	14.979	0.09	86	69.10
Cycloheptasiloxane, tetradecamethyl-	15.733	0.28	85	73.05
Apiol	17.712	0.23	76	222.00
Cyclooctasiloxane, hexadecamethyl-	18.199	0.23	85	73.05
9,12-Octadecadienoic acid, methyl ester	24.738	0.36	92	67.05
7-Octadecenoic acid, methyl ester	24.806	0.61	90	55.00

Table S4: Essential oil composition in Kala zeera (Kaksar Kargil collection)

Compound	Retention Time	Area%	Similarity	Base m/z
.alpha.-Phellandrene	6.923	0.05	85	93.10
.alpha.-Pinene	7.064	0.06	89	93.00
Bicyclo[3.1.0]hexane, 4-methylene-1-(1-methylethyl)-	7.829	0.40	95	93.05
L-.beta.-Pinene	7.903	0.15	93	93.05
.beta.-Myrcene	8.137	0.14	94	93.10
p-Cymene	8.767	12.44	96	119.05
D-Limonene	8.852	0.71	92	68.05
Trifluoroacetyl-.alpha.-terpineol	8.905	0.25	73	81.00
.gamma.-Terpinene	9.379	36.36	97	93.05
Bicyclo[3.1.0]hexan-2-ol, 2-methyl-5-(1-methylethyl)-, (1.a	10.089	0.09	85	93.05
Fenchol	10.354	0.06	86	81.10
3-p-Menthen-7-al	11.643	0.94	95	81.05
Cumic aldehyde	12.359	25.64	96	133.05
4-Isopropylcyclohexa-1,3-dienecarbaldehyde	13.038	2.33	94	79.05
1,4-p-Menthadien-7-al	13.104	19.47	95	79.05
Cyclohexasiloxane, dodecamethyl-	13.446	0.04	78	340.95
Caryophyllene	14.981	0.22	92	91.10
Cycloheptasiloxane, tetradecamethyl-	15.734	0.14	86	73.05
1H-Cycloprop[e]azulen-7-ol, decahydro-1,1,7-trimethyl-4-m	17.101	0.16	85	91.05
Caryophyllene oxide	17.212	0.06	75	79.10
Cyclooctasiloxane, hexadecamethyl-	18.197	0.10	86	355.00
Cyclononasiloxane, octadecamethyl-	21.123	0.07	79	72.95
1-Bromo-3,7-dimethyl-2,6-octadiene	26.920	0.10	75	69.10

Table S5: Essential oil composition in Kala zeera (Padder Valley Kishtwar collection)

Compound	Retention Time	Area%	Similarity	Base m/z
.alpha.-Phellandrene	6.918	0.12	94	93.05
.alpha.-Pinene	7.061	0.35	96	93.05
Bicyclo[3.1.0]hexane, 4-methylene-1-(1-methylethyl)-	7.824	0.53	96	93.05
.beta.-Pinene	7.897	0.70	97	93.05
.beta.-Myrcene	8.126	0.38	96	93.05
3-Carene	8.515	0.05	92	93.05
p-Cymene	8.763	14.27	96	119.10
D-Limonene	8.845	6.78	96	68.05
.beta.-Ocimene	9.166	0.18	94	93.05
.gamma.-Terpinene	9.377	36.42	97	93.05
Bicyclo[3.1.0]hexan-2-ol, 2-methyl-5-(1-methylethyl)-, (1.a	9.535	0.15	88	93.05
Cyclohexene, 3-methyl-6-(1-methylethylidene)-	9.904	0.12	95	93.05
Bicyclo[3.1.0]hexan-2-ol, 2-methyl-5-(1-methylethyl)-, (1.a	10.078	0.07	89	93.00
(3E,5E)-2,6-Dimethylocta-3,5,7-trien-2-ol	11.174	0.12	83	67.05
Perillyl butyrate	11.214	0.09	77	71.05
Terpinen-4-ol	11.390	0.23	89	71.05
.alpha.-Terpineol	11.590	0.06	86	59.05
3-p-Menthen-7-al	11.639	0.33	93	81.05
Cumic aldehyde	12.355	13.44	96	133.10
4-Isopropylcyclohexa-1,3-dienecarbaldehyde	13.032	1.38	94	79.05
1,4-p-Menthadien-7-al	13.099	24.15	96	79.05
(4aR,5R,9aR)-1,1,4a,8-Tetramethyl-2,3,4,4a,5,6,7,9a-octah	13.340	0.02	67	107.05
Caryophyllene	14.977	0.04	86	79.05
Cycloheptasiloxane, tetradecamethyl-	15.730	0.04	84	73.05

Table S6: Essential oil composition in Kala zeera (Atholi Kishtwar collection)

Compound	Retention Time	Area%	Similarity	Base m/z
Bicyclo[3.1.0]hex-2-ene, 2-methyl-5-(1-methylethyl)-	6.919	0.16	95	93.05
.alpha.-Pinene	7.061	0.73	96	93.05
Bicyclo[3.1.0]hexane, 4-methylene-1-(1-methylethyl)-	7.824	0.71	96	93.05
.beta.-Pinene	7.897	1.31	97	93.05
.beta.-Myrcene	8.126	0.39	96	93.05
p-Cymene	8.764	9.88	96	119.10
D-Limonene	8.851	0.71	93	93.05
.gamma.-Terpinene	9.382	40.66	91	93.10
3-p-Menthen-7-al	11.636	0.56	95	79.05
Cumic aldehyde	12.355	14.53	96	133.05
4-Isopropylcyclohexa-1,3-dienecarbaldehyde	13.029	1.96	94	79.05
1,4-p-Menthadien-7-al	13.102	27.51	97	79.05
Caryophyllene	14.978	0.61	95	91.05
(Z)-1-Methyl-4-(6-methylhept-5-en-2-ylidene)cyclohex-1-en	16.126	0.10	94	93.05
Geranyl isovalerate	26.904	0.17	77	69.05

Table S7: Essential oil composition in Kala zeera (Dawr Gurez collection)

Compound	Retention Time	Area%	Similarity	Base m/z
.alpha.-Phellandrene	6.917	0.09	94	93.05
.alpha.-Pinene	7.059	0.62	96	93.05
Bicyclo[3.1.0]hexane, 4-methylene-1-(1-methylethyl)-	7.823	0.50	95	93.05
.beta.-Pinene	7.896	1.51	97	93.05
.beta.-Myrcene	8.125	0.31	95	93.05
p-Cymene	8.762	18.76	96	119.10
D-Limonene	8.849	0.66	90	93.05
Eucalyptol	8.905	0.13	69	71.05
.beta.-Ocimene	9.165	0.16	95	93.10
.gamma.-Terpinene	9.377	40.22	96	93.05
Cyclohexene, 1-methyl-4-(1-methylethylidene)-	9.902	0.14	96	93.10
(3E,5E)-2,6-Dimethylocta-3,5,7-trien-2-ol	11.172	0.16	89	67.00
(+)-cis-Verbenol, 2-methylpropionate	11.215	0.12	78	71.00
Terpinen-4-ol	11.388	0.18	89	71.05
3-p-Menthen-7-al	11.636	0.36	95	79.05
Cumic aldehyde	12.353	16.72	96	133.05
4-Isopropylcyclohexa-1,3-dienecarbaldehyde	13.030	1.83	94	79.05
1,4-p-Menthadien-7-al	13.098	17.38	96	79.05
Caryophyllene	14.977	0.07	92	93.05
Cycloheptasiloxane, tetradecamethyl-	15.731	0.09	87	73.05
